# The Christian Science Deception

**Published:** 1899-08

**Authors:** 


### THE CHRISTIAN SCIENCE DECEPTION.

CHRISTIAN science and its adherents came out into the open at the hearings before the committee on ordinances of the board of aldermen recently, when the ordinance proposed for the regulation of promiscuous treating of the sick and injured was under consideration. This ordinance was introduced at Dr. Wende's request. Briefly, it requires that a report of all contagious cases under treatment be made to the Health Department within a certain number of hours. This includes christian scientists, faith curists, mesmerists, clairvoyants, long-distance practitioners and other curiosities.

Naturally, the christian scientists, who do not diagnosticate disease because they do not believe in disease and pray over diphtheria and scarlet fever with the same degree of fervor as they exhibit in curing corns, sounded the alarm and flocked to the City Hall to fight the measure. They brought their "healers" and their friends and their dupes. They all had much to say, and some of the women of Buffalo—some of them prominent as prominence is gauged on the social scale nowadays—rushed into print to give vent to their excess of feelings over the outrage which a health commissioner of the modern school was inflicting upon the annointed of the Lord. One woman in particular, whose husband had "passed on" while prayers were being said for his relief, went so far as to publicly state that in the event of the passage of the ordinance she would break the law just as often as she could. Well and good. That is her privilege. If she does, however, the JOURNAL feels confident that the lady will eventually be in a position to speak from the depths, and that her social position will in no wise be a safeguard for her; that she will meet the same fate as an ordinary, every-day and less pretentious violator of the health ordinances.

At the hearings Dr. Wende made his position very clear. He showed how dangerous was the practice of permitting these "healers" to trot about from one case to another; from scarlet fever, diphtheria and other communicable diseases, to whooping cough and cramps, and then to the fountain head of their alleged inspiration, carrying and disseminating the germs of disease. He showed how futile it would be to attempt to prevent epidemics if an irresponsible body of fanatical believers were permitted to treat diseases which actually existed, but which they in their blind confidence in the soothsayings of an acute seeress deny as ever existing, either in fact or fancy.

The "healers" had a chance to talk, and they did talk. They talked. That is their profession and their therapeutics. That's all they did do, save in one case, where a very brave gentleman, whose El Mahdi-like devotion led him to make a blatant fool of himself, asserted that he could cure anything from croup to corns and back again through the long list of human ills, chronic and acute.

Dr. Wende made a proposition. He offered to supply a certain number of chronic cases from his own practice and of his own observation. If cures were effected—he, Dr. Wende, would then become a christian scientist.

The scientists claim they are not amenable to the law because they do not give medicine. It seems to the JOURNAL that any one who pretends to or actually does relieve pain, cure the sick and make the weak strong, and does this for pay, practises the art and science of medicine, no matter whether he administers drugs, lays on hands, gives advice, or prays. The ultimate object is the same in every case—the relief of suffering.

The whole object of Dr. Wende's crusade is apparent to every sensible human being, who is not blinded by bigotry or filled with a fixed fanaticism. No one objects to a grown up person enjoying personal liberty to its fullest measure. No one objects to a grown up person employing any means which seem to him proper to reach the Throne of Grace, nor to his employing any means to gain relief from pain or suffering. What is objected to, however, is the practice of submitting babies and over-awed children to the muttering of prayers by christian science healers, the hanky-panky finger weaving of faith curists, or the snake-skinning, stick-throwing incantations of voodoos and witch-doctors.

The law steps in and takes charge of a minor child's material interests; it should also, and does if properly interpreted, step in and guard his physical interests with equal fidelity and watchfulness. If adult believers prefer to "pass on" to prayer accompaniment, all well and good. There is none to say them nay; but they have no moral or legal right to interpret in its literal sense that most comforting of all Biblical passages: "Suffer little children to come unto Me, for of such is the Kingdom of Heaven."

